# Facilitators and barriers to deferring imaging for acute low back pain: a qualitative study

**DOI:** 10.1186/s12875-025-02902-1

**Published:** 2025-07-02

**Authors:** Carly A. Robinson, Melissa M. Gosdin, Camille S. Cipri, Ilona Fridman, Gary Weinberg, Anthony Jerant, Andrew Hudnut, Joshua J. Fenton

**Affiliations:** 1 School of Medicine, University of California, Davis, Sacramento, CA USA; 2 Center for Healthcare Policy and Research, University of California, Davis, Sacramento, CA USA; 3 Hackensack Meridian Health School of Medicine, Nutley, NJ USA; 4 Georgetown University Comprehensive Cancer Center Lombardi, Washington, DC USA; 5 Sutter Institute for Medical Research, Sacramento, CA USA; 6 Department of Family and Community Medicine, University of California, Davis, 4860 Y Street, Suite 2300, Sacramento, CA 95817 USA

**Keywords:** Qualitative studies, Doctor-patient communication, Imaging, Low back pain, Low-value healthcare

## Abstract

**Background:**

Early imaging for uncomplicated acute low back pain has no diagnostic benefit yet is completed after nearly one-quarter of primary care visits for acute back pain. This qualitative study examined patient and clinician perspectives on facilitators and barriers to deferring imaging for acute low back pain, including potential messages regarding a watchful waiting strategy without early imaging.

**Methods:**

Qualitative data derived from six patient focus groups (*N* = 30 patients with recent visits for acute low back pain) in Sacramento, CA and nine semi-structured physician interviews in 2020. Patients were asked about expectations regarding imaging, perceptions of care received for acute low back pain, and perspectives about potential messages encouraging a watchful waiting approach without early imaging. Clinicians were asked about facilitators and barriers to deferring low-value imaging. We used thematic analysis guided by grounded theory to identify and integrate themes.

**Results:**

Over half of patients had received early imaging during their recent back pain episode. Patients expected physicians to provide a detailed rationale for ordering imaging or not. Patients were typically not persuaded by information on potential harms of imaging and sometimes thought discussion of imaging harms would undermine their trust in the clinician. Patients would be more willing to defer imaging if provided detailed and empathic guidance on pain management. Physicians expressed confidence in advocating a watchful waiting approach without imaging but acknowledged challenges in building patient trust during time-pressed visits, particularly when seeing patients for the first time.

**Conclusions:**

This qualitative study highlights several challenges to deferring early imaging in acute low back pain, as patients typically expect early imaging and were skeptical of clinician messaging about imaging harms. Physicians highlighted lack of a previously established, trustful relationship as a common structural barrier to deferring low-value spinal imaging.

**Supplementary Information:**

The online version contains supplementary material available at 10.1186/s12875-025-02902-1.

## Introduction

Acute low back pain is one the most common reasons for primary care visits [1] and a major worldwide cause of disability [2]. Common approaches to the initial evaluation and management are often either ineffective or iatrogenic, which has prompted a call to action from the *Lancet* Low Back Pain Series Working Group to improve the primary care evaluation of low back pain [3].

Uncomplicated acute low back pain, or pain that lacks high-risk features suggestive of an underlying medical etiology, has a favorable prognosis, and early lumbar spinal imaging generally yields no diagnostic benefit [4]. However, early imaging occurs in one in four patients presenting to primary care with acute low back pain [5]. Potential risks of early spinal imaging include false-positive or incidental findings leading to higher rates of unnecessary interventions or surgery [6], patient anxiety [7, 8], and radiation exposure [5, 9]. Hence, early imaging for acute uncomplicated low back pain is a target for reducing unnecessary costs and potential iatrogenesis of initial back pain evaluation. Reduced early imaging for acute uncomplicated low back pain has been endorsed as a quality process measure [10], and the *Choosing Wisely* campaign prioritizes the reduction of early imaging for low back pain through recommendations of several primary care societies internationally [11]. Specifically, the *Choosing Wisely* campaign recommends that imaging is not performed for low back pain within six weeks of onset, unless concerning symptoms such as severe neurological deficits are present or there is otherwise concern for a serious etiology [11].

Previous qualitative studies point to several patient and clinician factors that may challenge efforts to reduce early imaging for acute low back pain. In a scoping review of low back pain patients’ perceived needs for medical services, patients broadly expected to receive imaging and believed it could be both reassuring and confirm the diagnosis [12]. Meanwhile, qualitative and quantitative studies of clinicians have found several factors that lead clinicians to order imaging, including patient requests or pressure to obtain imaging [3, 13–16], the perception of having insufficient time to counsel against imaging [14–16], clinicians’ beliefs that normal scans will reassure patients [16], and worries about missed diagnoses leading to litigation [14, 15]. Relatively few prior studies have focused specifically on imaging for acute (rather than subacute or chronic) back pain, and to our knowledge, none have sought both patient and clinician input on specific messaging to foster patient acceptance of deferred imaging.

A watchful waiting approach is one potential management strategy that may reduce early imaging in low back pain. A clinician pursuing a watchful waiting approach implements a conservative treatment plan during a period of observation and only pursues imaging if the patient does not improve during this period. Watchful waiting has been shown to result in less ordering of low-value diagnostic tests for non-specific symptoms [17] and fewer antibiotic prescriptions for pediatric ear infections [18]. In preparation for a randomized trial of a communication intervention to increase adoption of a watchful waiting approach to low back pain in primary care (NCT 04255199) [19], we conducted a qualitative study to identify patient and clinician facilitators and barriers to deferring early, low-value imaging for acute back pain with a specific focus on communication strategies and messages to foster a watchful waiting approach.

## Methods

### Design, setting, and sample

We used focus groups of patients with recent visits for acute back pain and key informant interviews of expert clinicians to identify major themes and subthemes. We recruited a purposive sample of patients who had sought care for acute low back pain using poster flyers at primary care clinics on the University of California, Davis Medical Center campus in Sacramento, CA, and online advertisements. Patients were eligible if they were aged ≤ 65 years, were English-speaking, and had seen a physician for acute low back pain in the last two years. Patients were excluded if they had a history of spinal surgery or chronic back pain. After patients contacted staff expressing interest, staff confirmed eligibility and obtained self-reported demographic data prior to scheduling focus groups.

We purposively recruited nine practicing physicians to participate in key informant interviews. We prioritized recruiting active primary care clinicians who would have experience and insights regarding patient responses to alternative management strategies in acute back pain, including five primary care physicians in active clinical practice and four experts in drivers of low-value testing or patient-doctor communication (a family physician, two general internists, and a hematologist). The University of California, Davis Institutional Review Board approved the study. For both patient focus groups and clinician interviews, we continued recruitment and data collection until thematic saturation was reached.

### Data collection

The investigators developed original questions to guide semi-structured interviews of patients during focus groups and clinicians during telephone interviews (Supplemental Appendix). An experienced qualitative researcher (MG) conducted six virtual patient focus groups (3–6 patients per group; 30 patients total) from April-June 2020. Focus groups lasted approximately 90 min, were audio-recorded and transcribed by a professional service. During focus groups, patients were asked about their experiences with low back pain, obtaining medical care, and their expectations regarding the diagnosis and management of low back pain, including imaging. The moderator explored patients’ receptivity and reaction to potential clinician messages communicating a watchful waiting recommendation regarding imaging. These messages included an expression of optimism and confidence about the favorable prognosis of uncomplicated low back pain, the diagnostic limitations and potential downsides of early imaging, and an expression of willingness to pursue imaging later if low back pain worsened or did not improve. Data on whether patients had previously received imaging for acute low back pain was verbally obtained during focus groups, and the moderator probed for how clinicians’ communications about deferring imaging affected patient trust.

The same qualitative researcher also conducted telephone interviews with nine clinicians from January-October 2020 to elicit feedback on communication strategies that the clinicians used to facilitate conservative care without low-value imaging in patients with acute back pain. Interviews lasted 30–60 min, were audio recorded, and transcribed.

### Analysis

We conducted a thematic analysis with tenets of grounded theory approach to identify themes from the focus group and interview data [20]. Three trained coders (MG, CC, GW) began with an initial review of the focus group and clinician interview transcripts and applied open coding and analytical memoing [20, 21]. The team approached coding and analysis iteratively, meeting regularly to discuss findings and resolve discrepancies. After reaching team consensus on broad coding categories, specified a priori or emergent from the data, we developed a codebook, and two coders independently coded each transcripts using Dedoose qualitative software. The codebook was revised as needed during the coding process [22]. Upon completion of coding, we identified themes arising from both the patient focus groups and physician interviews. The COREQ checklist was consulted in the reporting of this study [23].

## Results

The patient sample was socioeconomically diverse, and over half reported having received imaging during acute back pain episodes (Table 1). The thematic analyses revealed six themes (Table 2).

**Table 1:** Focus group patient characteristics (*N* = 30)

Characteristic	*n* (%)
**Age**,** mean (range)**	38 (23–60)
**Female sex**	20 (67)
**Hispanic/Latino Ethnicity**	6 (20)
**Race**	
White	19 (63)
Asian	4 (13)
Black	6 (20)
Other	1 (3)
**Education**	
Any graduate level education (e.g. Master’s or PhD courses OR degrees)	3 (10)
Graduated college (received degree)	19 (63)
Some college (did not graduate)	6 (20)
High school or less	2 (6)
**Annual Household Income**	
Less than $20,000	2 (7)
$20,000 to less than $35,000	5 (17)
$35,000 to less than $75,000	6 (20)
$75,000 to less than $125,000	13 (43)
Over $125,000	4 (13)
**Marital Status**	
Never married	7 (23)
A member of an unmarried, committed couple	4 (13)
Married	14 (47)
Separated/Divorced	5 (16)
**Health Status**	
Very good	17 (57)
Good	9 (30)
Fair	4 (13)
**Insurance Status**	
Private Insurance/Health Maintenance Organization	19 (63)
Any Medicaid	5 (16)
Medicare	2 (7)
No insurance, other, or unsure	4 (13)
**Doctor ordered X-ray**,** MRI**,** or CT-scan to evaluate your back pain**	16 (53)

**Table 2:** Themes regarding deferring imaging for acute back pain

Themes	Representative Patient Quotes	Representative Clinician Quotes
1. Perception of Early Imaging as the Standard of Care	“It just feels like I didn’t get complete treatment because I’m still suffering from the pain. I wish [the doctor] would have at least offered to do some scans just to feel like I was getting everything that could possibly help or solve a problem, I guess.” (Focus Group #3)	“You have to give [patients] some reasonable rationale as to why no imaging now. Because it’s one of those things like… you don’t want to give people the impression that we’ll try generic treatment, and if the generic treatment doesn’t work then you get the imaging, you get specialized treatment… because everyone wants their care personalized.” (Clinician #8)
2. Imaging as a Means of Alleviating Patient Anxiety and Fear	“I would say that for me… [imaging] was offered… in a wishy-washy way… again, I didn’t think I needed it. But I think the benefit of having it would have been just a lot of peace of mind… knowing that it’s not a big structural problem would have been… a huge relief.” (Focus Group #6)	“Patients don’t like sitting with an unknown. The anxiety of the unknown is horrendous. They just want to know. Even if it’s not going to fix anything, [they want to] have the MRI just to know, just because ‘I want to know.’ That’s a really hard conversation to have.” (Clinician #5)
3. Skepticism Regarding Potential Negative Consequences of Imaging	“[In] my opinion, there’s not a whole lot of negativity to [imaging]. I see no reason not to do it if we could.” (Focus Group #4)	“I will talk about the downside. I’ll say, ‘People think that it’s just better to know, but oftentimes, imaging will show things that actually aren’t going to impact your health. But once we see them on the imaging, we end up doing more tests and sending you down this garden path of getting stuff done that doesn’t improve your health. And it’s really just a burden.’” (Clinician #6)
4. Distrust Engendered by Messages to Defer Imaging	“I don’t trust my doctor because… I feel like I’m just being given painkillers, or medication, when I want to know more of what my problem is, how can we fix it?” (Focus Group #2)	“It’s clearly easier to do this if it’s a patient you have a relationship with, who trusts you already. Trying to create that trust… Like if it’s the first time you’re meeting that patient, it’s really hard to create trust while also not giving them what they want.” (Clinician #6)
5. Importance of Understanding Patient Expectations	“Personally, I expected them to do imaging… But then when they didn’t see anything on the [imaging] findings, they were like, just do some PT. You prescribe PT for everything, buddy. Can’t I get a shot? Don’t I get an injection? Is there something else we can do about this?” (Focus Group #1)	“Some patients coming into the office are looking for something that they can’t do for themselves, and that something could be reassurance, an exercise program… any number of things. I try to see what it is that someone wants and what approach they want, what their goal is, and then I try to see whether that request makes sense in terms of the goal that they have.” (Clinician #3)
6. Importance of an Empathic Pain Management Plan	“The expectation that I had was to be referred to a massage therapist or physical therapist and to get direction. I didn’t want to get shots and stuff like that, but I wanted to get direction on what to do while being able to help myself as well.” (Focus Group #1)	“I personally will never tell anyone to just wait… Six weeks is a long time to just sit there, you have to do something… [Whether] it’s a muscle relaxant, heat/ice, at-home exercises, formal physical therapy, massage, acupuncture… You have to give them some plan.” (Clinician #8)

### Perception of early imaging as the standard of care (theme 1)

Patients in several focus groups expected imaging and viewed its exclusion as a potential indicator of lower care quality. These patients felt that imaging was “the bare minimum” (FG5) and otherwise felt that they “didn’t get a complete treatment” (FG3) without it. They sometimes believed that they were being denied resources if not offered imaging: “Are they depriving me from these types of resources, and why would they do that?” (FG3).

Physicians acknowledged that many patients expected imaging as an indicator of quality care. One noted that patients often feel like “they must need imaging” (C4) when in severe pain, highlighting the importance of being “thorough and careful and empathetic [in order to avoid being seen as] somebody who’s just trying to deny resources to a patient who is in pain” (C7).

Patients consistently expressed a desire for an explanation from clinicians regarding whether imaging should occur and when. However, some expressed an openness to a watchful waiting approach if clinicians transparently communicated a rationale for deferring imaging:

“I think the doctors should maybe explain [their rationale] to the patient. Because… if I didn’t know much about back pain and they told me, ‘Oh, we’ll just wait on this,’ then I’m going to feel like, ‘Oh, you’re not going to take care of my issue that I came in here for?’ But if they thoroughly explained it to me, then I would understand more” (focus group #1; FG1).

When clinicians did not explain their rationale, patients reported feeling that clinicians were either rushed or uncaring. In contrast, patients thought a thorough explanation was indicative of the clinician’s concern and care for their pain.

Physicians also emphasized the importance of explaining that imaging could be revisited at a later visit:

“If [conservative management] doesn’t work… then we can consider other steps, and that may include imaging, or seeing a specialist, or something along those lines. That way they can see that this… is the initial step, and if this doesn’t work, there is a back-up plan” (clinician #5; C5).

### Imaging as a means of alleviating patient anxiety and fear (theme 2)

Patients frequently desired imaging as a means of assuaging anxiety and providing reassurance. Patients who received imaging for acute back pain noted “the pain didn’t go away but [the MRI] made me feel calmer” (FG4) and imaging provided “a huge relief” (FG6) in ruling out serious causes of back pain. Nevertheless, although over half of patients received imaging, few felt that it was definitively helpful: “I don’t know if it really helped or not. I mean, maybe for… peace of mind, knowing that the doctor is actually doing something, but it didn’t show anything” (FG2).

Several physicians noted that patient anxiety is a major barrier when trying to persuade patients to defer imaging. Physicians reported that some patients believe that “imaging itself would [be] therapeutic” (C1) and “that by getting an MRI scan, somehow they would feel better” (C3). Hence, physicians reported instances where they had ordered imaging primarily to address patient anxiety:

“There are some patients who are so anxious… that I deem it worthwhile to do some testing that may help to alleviate that. But I would say it’s pretty rare, because if a patient is convinced they have cancer and their CT scan is normal and they want a MRI and the MRI’s normal, they’ll want a PET scan, they’ll want something else. So that’s a trap” (C3).

However, physicians also noted that spinal imaging may worsen anxiety due to the presence of incidental findings:

“Sometimes [scans] might provide some reassurance, but then other times they provide anxiety and headache. And the reason is you may find something like an incidental finding… Usually it’s nothing, but because… you can’t explain it, you have to repeat the imaging six months later… So sometimes they can reassure, but… there’s also psychological harm that can come from it” (C7).

### Skepticism regarding potential negative consequences of imaging (theme 3)

Patients expressed openness to physicians discussing possible negative consequences of imaging (i.e., false-positive or incidental findings, radiation). However, patients largely responded skeptically to specific messaging about these consequences and felt that potential downsides would not dissuade from receiving early imaging.

Patients were less receptive to discussing the possibility that false-positive results may lead to unnecessary interventions or surgery. Participants in some focus groups were skeptical that current imaging technology might produce false-positive results, while others did not understand how patients who received imaging might be more likely to receive further diagnostic imaging or referral for interventions, such as biopsy, injections, or surgery.

Patients understood the meaning of incidental findings but had differing opinions about whether they were harmful. One patient stated they “would have been happy” if their back imaging “caught [an incidental finding] before it got worse” (FG1). However, another patient felt that “if they’re not going to do anything about [incidental findings], don’t tell me about them” (FG1).

The risk of radiation associated with x-ray and CT imaging was familiar to patients and was thought to be the most obvious negative impact of these imaging modalities. When discussing whether doctors should explain the radiation risks, one patient stated, “that actually is what I want to hear, the transparency, the honesty… I don’t want to be putting myself into more harm” (FG1). However, most focus groups felt that the benefits of occasional imaging outweighed the risk of radiation, especially during an acute back pain episode: “I feel like if you get it once it’s not going to kill you. Obviously, it’s not good for you… but when you’re trying to relieve this pain, I feel like it’s important to get [imaging]” (FG3).

Physicians were divided on whether they believed patients would be responsive to discussions of possible harms from imaging. Physicians who did not routinely discuss potential harms felt that “it’s not what [patients] want to hear” (C5) and sometimes makes patients “feel like you’re coming up with an excuse to not give [them] the imaging [they] want” (C4). However, other physicians felt that “focusing on the risk of patient harm is a useful tool to discourage a patient from wanting a more aggressive diagnostic approach” (C7).

### Distrust engendered by messages to defer imaging (theme 4)

While patients in most focus groups were open to discussing possible negative consequences of imaging, some stated that clinicians raising this topic sparked distrust. One patient felt as if his physician was “talking down to [him and] trying to save money” (FG4) when discussing potential harms of imaging. Patients stated that when physicians did not meet their expectation for imaging, they were “not taken that seriously” (FG3), were “just a chart” (FG3), or were “brushed off” (FG5). Patients were more likely to trust their physician’s recommendations when they had a preexisting patient-doctor relationship: “I’ve been with this doctor a while and I really trust his judgment and diagnosis in the past, so I had no reason not to be comfortable [with their recommendation to defer imaging]” (FG3).

Physicians echoed this sentiment, stating that their patients were more likely to agree with a conservative management approach if they had a preexisting relationship, although frequently they are scheduled to see patients for acute back pain who they have not previously met: “If it’s the first time I am meeting that patient, it’s really hard to create trust while also not giving them what they want” (C6).

Some physicians also touched on the concept of patient satisfaction, stating that occasionally they had ordered imaging because they felt “it’s not worth hurting the doctor-patient relationship by saying no” (C8). Some physicians raised the cost of imaging with patients, but overall physicians felt that this was a less effective discussion point and had the potential to undermine rather than enhance patient trust.

### Importance of understanding patient expectations (theme 5)

Patients reported a variety of motivations for seeking care, such as relieving pain or obtaining reassurance. Physicians recognized that patients may have different motives for seeking care, and therefore believed it was important to elicit and address patient expectations: “I think the provider really needs to assess… what they’re actually concerned about… Once you can diagnose what the concern is, then only can you really build that trust with your patients” (C2).

Physicians generally opted to directly ask patients what they were most worried about or what expectations they had for their appointment. Once expectations were understood, physicians often felt the need to manage unrealistic expectations:

“[Some patients] come and their expectations are really different from reality and they get very frustrated. So, I try to give people some expectations… But it’s hard, I’m just one person and I have a certain amount of time and I can’t, it’s almost like everybody needs a course on how to manage their own health” (C4).

### Importance of an empathic pain management plan (theme 6)

Patients expressed the need to be heard and receive clinician empathy. Patients felt that their physicians cared about them when they “gave their full attention, actively listened, asked follow-up questions” (FG5) and showed “compassion and empathy” (FG4).

Physicians discussed the value of “listening to the patient and conveying that you hear how much they’re suffering… You’re not blowing off their complaint. You’re acknowledging that this is really difficult and that they’re in a ton of pain” (C6). By validating patients’ concerns, physicians felt that they were better able to build trust and demonstrate that they were “on the same side as the patient” (C6) in wanting to help address their pain and concerns.

Many patients stated that they went to their doctor seeking expertise and were disappointed when they did not receive a conclusive diagnosis or solution for their pain. Patients were also displeased when their physicians recommended measures they were already trying at home, such as taking over-the-counter pain medications:

“I was expecting more expertise because really, I was given, like, ‘Take ibuprofen and wait.’… I could have told myself that, and that’s what I’ve already been doing. So, I was expecting a bit more knowledge” (FG6).

When patients felt that they had not received adequate pain management instructions from their doctors, they often conducted their own research and utilized acupuncture, yoga, or online exercises:

“I wasn’t offered any exercise [or] physical therapy. I wasn’t offered imaging. It’s like, again, just felt like it was a waste of my time… Well, at that point, I found the Google exercises to lay on the floor, pull your knees up to your chest and found different exercises to do. And I started alleviating the pain on my own and decided… maybe I should just stay away from the doctor for a while” (FG1).

Patients reported being more likely to accept a watchful waiting approach when doctors provided suggestions for pain management and expressed that imaging could be explored later if conservative measures failed. Physicians were aware of this and stressed the importance of providing treatment options, including over-the-counter analgesics, heating pads, lidocaine patches, home exercises, and physical therapy. Physicians recommended informing patients that there are “other tools in [the] toolkit if these don’t work” (C9), such as repeat exams, imaging, or pain specialist referrals. Overall, physicians felt that, while some patients specifically wanted imaging, many simply wanted to feel that they were “doing something about the problem” (C6) as opposed to just waiting.

## Discussion

This qualitative study extends prior research on patient and clinicians’ perspectives on the role of imaging in low back pain evaluation with new insights regarding patients’ skepticism regarding the risks and limitations of imaging and mistrust engendered by potential watchful waiting messages. Consistent with prior qualitative and quantitative studies [12, 16, 24], while many patients in our focus groups expressed openness to the concept of a watchful waiting approach, they also often expected to receive imaging and viewed it as an indicator of high-quality care. Patients and clinicians perceived that careful and empathic communication in the context of a trusting relationship would facilitate deferral of low-value spinal imaging.

Our study highlights the importance of patient trust as the foundation for patient acceptance of clinicians’ advice to defer imaging, yet clinician messages emphasizing the limitations and risks of imaging were viewed skeptically and engendered mistrust. This finding is consistent with a qualitative study that found that a trusting patient-doctor relationship is the most influential factor in whether patients accept *Choosing Wisely* recommendations [25]. At the same time, many patients may view clinician suggestions to defer or not obtain tests or treatments as denials of care that might be related to insurance restrictions or clinicians’ potential conflicts of interest. As an outgrowth of *Choosing Wisely*, the American Board of Internal Medicine Foundation launched the *Building Trust* initiative, which aims to identify and promote methods for building trust in patient-doctor relationships [26]. Patients mistrust of watchful waiting messages may be particularly pronounced in the United States context, where patients may perceive primary care clinicians as “gatekeepers” and care may be more fragmented than in health systems in other developed countries [27].

Study physicians noted the heightened challenge of advocating for watchful waiting when seeing a patient for the first time. Similarly, a qualitative study on continuity of care found that patients’ trust was easily undermined on a first encounter with a new physician, whereas an established relationship fostered patient trust [28]. Despite the recognized benefit of continuity of care, patients are less likely to see their primary care physicians than other clinicians for acute care visits in the United States [29], which may be a pervasive structural barrier to disseminating a watchful waiting approach in this context. As in prior studies [14–16], clinicians perceived limited time during encounters as an environmental barrier to engaging patients in detailed conversations about the limited benefits and non-negligible risks associated with early imaging.

Clinicians also acknowledged patient anxiety as a barrier to deferring imaging, as several clinicians believed many patients requested imaging seeking reassurance, consistent with prior qualitative studies [12, 15, 16]. However, research has shown that diagnostic tests have limited efficacy in reassuring to patients [17, 30], and patients in our focus groups had mixed opinions on whether imaging relieved their anxiety, as some had incidental findings that resulted in unnecessary stress. Other studies have similarly noted that patients may experience distress or frustration as a result of inconclusive or negative imaging results, respectively [7]. Together, these findings suggest the need for brief interventions that primary care clinicians can employ to ameliorate patient fear and anxiety without ordering unnecessary diagnostic tests. The clinician intervention developed based on the current qualitative study emphasized optimism, reassurance, trust-building, and empathic communication [31]. Although the intervention showed promise in boosting empathic communication, it did not reduce early imaging in patients with acute low back pain, suggesting the need for systems-level interventions to mitigate early imaging [32].

Study patients were generally not persuaded by the potential risks associated with low-value imaging. While patients thought that the risk of radiation merited discussion, they generally thought that radiation risks were inconsequential compared to the potential benefits of discovering the source of their pain. Discussions of false-positive results resulted in patient skepticism, despite lumbar MRIs possibly having a false-positive rate as high as 81% [33]. Prior qualitative studies noted similar sentiments, with patients believing that the usefulness of imaging outweighed potential harms such as radiation or time inconvenience [24] and community members expressing skepticism at messaging related to risks of imaging [34]. Patients in our study also considered it far-fetched that imaging might lead to a higher chance of undergoing invasive tests or treatments.

Our study may be limited by non-representativeness of the patient or physician samples, although we judged the transferability of the findings to be high for primary care practices both in and outside the United States. Although we achieved thematic saturation in the focus groups and interviews, additional themes may have been revealed had we collected additional data from larger patient or physician samples, as this study included a total of 30 patients and nine physicians. While we recruited patients with recent visits for acute back pain, some patients may have had a more chronic course with a recent acute pain episode. Although all patients were seen within the past two years for evaluation of acute low back pain, patients who were seen earlier within that time frame may recall their experiences differently than those who were seen more recently. The study was also conducted during the Covid-19 pandemic, although we doubt the pandemic would have altered patients’ and clinicians’ perspectives on early spinal imaging for low back pain.

## Conclusions

In this qualitative study, we found that patients with acute low back pain often expected to receive spinal imaging and were often skeptical about potential clinician messages addressing the limitations and potential harms of early imaging. Physicians in our study expressed confidence in advocating a watchful waiting strategy for acute low back pain with deferred imaging but acknowledged challenges of building trust with unfamiliar patients and finding time for empathic and thorough communication. Our findings highlight the need to facilitate trustful clinical conversations in primary care that lead to evidence-based testing and treatment for patients with acute low back pain.

## Electronic supplementary material

Below is the link to the electronic supplementary material.

Supplementary Material 1

## Data Availability

The data that support the findings of this study include anonymized transcripts of patient focus groups and clinician interviews. These transcript data are available from the authors upon reasonable request.

## Declarations

### Prior presentation

A version of this paper was presented orally at the North American Primary Care Research Group annual meeting in Quebec City, QC, Canada, November 23, 2024.

## Abbreviations

C: Clinician
FG: Focus group
