# Supplementary material for: Facilitators and barriers to deferring imaging for acute low back pain: a qualitative study
Source: BMC Prim Care. 2025 Jul 2;26:204. doi: 10.1186/s12875-025-02902-1 (PMC12220053; doi:10.1186/s12875-025-02902-1)
Supplement: Supplementary file 1 — Supplementary Material 1 [file 12875_2025_2902_MOESM1_ESM.pdf]

SUPPLEMENTAL APPENDIX

"Facilitators and Barriers to Deferring Imaging for Acute Low Back Pain: A Qualitative Study"

Qualitative Interview Guides for Patient Focus Groups and Clinician Interviews

I. Interview Guide for Patient Focus Groups

General Introduction & Verbal Consent

The purpose of this study is to learn more about patients' experiences with low back pain and getting care for their back pain. Using what we learn, we hope we can educate health care providers on the best way to understand patients' expectations for the evaluation of their back pain and how to communicate effectively with their patients with back pain.

The focus group will last approximately 60 minutes. If there are any questions you do not want to answer, you are free to skip them. You are also free to end the interview at any time.

The **interview will be recorded and transcribed** so we can review the conversation after the interview. Only the evaluation team will review the transcripts. Your responses are **confidential**. You will not be identified in any description or summary of the results.

The interview will be conducted as a guided conversation. I have a list of topics and general questions that I will go through, but your responses will guide the interview.

Thank you again for your participation. Does what I outlined sound okay? Do you have any questions before we begin? **START RECORDING**

Guiding Questions

When you sought care for your back pain, what were your primary goals?

When you saw your doctor for low back pain, what was more important to you: reducing and managing the pain, or making sure that you did not have a serious problem that would lead to long-term pain or disability?

1. When you sought care for your back pain, what were your expectations from your health care provider?
    a. *Probe for specific experiences that were particularly helpful or unhelpful during their visit with their health care provider.*

What did you expect from your provider in terms of physical examination?

What did you expect from your provider of diagnostic test, such as blood tests, x-rays, or scans?

How did your doctor talk to you about the need for diagnostic tests, such as x-rays or scans, for your back pain?

How many of you received an x-ray or scan and did it make a difference to you in terms of taking care of your back pain?

Do you also think it helped your doctor in developing a treatment plan for you?

If your doctor did not order a xray or scan, how did they explain the reason they did not recommend x-rays or scans? Were you satisfied with their explanation? *Probe for issues of trust.*

Now we'd like to share with some information about clinical guidelines for the diagnosis and treatment of low back pain.

First, guidelines note that back pain has very favorable prognosis. That is, that large majority of patients with back pain will have complete resolution of their pain within weeks. Most patients' pain is caused by strain of muscles or ligaments in the spine that heal well with minimal intervention even when back pain is severe. Except in unusual cases, guidelines do not recommend x-rays, CT, or MRI scans in the first few weeks of an episode of back pain. Indeed, guidelines specifically advise doctors not to order x-rays or scans during this period due to the risks of false-positive results that might promote unnecessary further evaluation or even unhelpful intervention, such as spinal surgery. False-positive results mean that the x-ray or scan shows something that looks like it might be causing back pain when in reality the x-ray or scan abnormality is really causing any pain or back problem. Despite these clinical guidelines, about one-third to one-half of back pain patients received x-rays or scans.

We're interested in your perspectives as patients about these clinical guidelines.

What are thoughts about the recommendation that doctors should not order xrays or scans within the first month of a back pain episode?

What would need to hear from your doctor to accept their recommendation not to order xrays or scans?

At this point, we'd like to introduce you to the idea of "watchful waiting." Watchful waiting refers to when a doctor does not immediately investigate or treat a problem but observes carefully for a period of time before deciding whether to test or begin treatment. In a patient with back pain, for example, the doctor might recommend a trial of some stretching exercises

and over-the-counter pain medication for a period of two to three weeks. If the pain were to persist or increase beyond that time, then the doctor might consider an xray, CT, or MRI scan. This would be an example of "watchful waiting" for low back pain.

After hearing about the idea or concept of "watchful waiting," do you find the idea acceptable or appealing and why?

Would you have any concerns if a doctor were to suggest to you a watchful waiting approach if you had an episode of low back pain?

What information could your health care provider share with you to reassure you that watchful waiting is an acceptable approach for your back pain?

Share example scenarios such as possible false-positive findings, unnecessary treatments, including spinal surgery, etc. and elicit feedback.

1. Now I'd like to share some "watchful waiting" messages health care providers might say to a patient with back pain during a visit and ask you all to comment on what you find appealing or unfavorable about these. [Examples will be drafted based on pre-RCT key informant interviews with clinicians]
    a. *Based on feedback, probe for reasons for reactions and how the messages might be improved so patients are more receptive.*
2. "I don't recommend a scan or xray now but it may be a good idea to get one if your symptoms persist or worsen over the next three or four weeks."

    "I'm concerned that an xray or scan will put you at risk of false-positive tests and unnecessary surgery."

    "I'm afraid an xray or scan may actually slow your recovery, as xray abnormalities can sometimes convince people that they have a 'bad back' even though such abnormalities are very common even in patients without back pain."
3. Is there anything else you would like to add to our discussion?

Thank you all for your time!

II. Interview Guide for Clinician Interviews

General Introduction & Verbal Consent

The purpose of this study is to see if an educational intervention directed at clinicians can increase their delivery of a watchful waiting message to patients requesting low-value spinal imaging. I will ask questions about your experience with patients who request low-value imaging and barriers and facilitators to patient acceptance of a watchful waiting strategy. I will also ask for your input on the educational intervention.

The interview will last approximately 45 minutes to an hour. If there are any questions you do not want to answer, you are free to skip them. You are also free to end the interview at any time.

The **interview will be recorded and transcribed** so we can review the conversation after the interview. Only the evaluation team will review the transcripts. Your responses are **confidential**. You will not be identified in any description or summary of the results.

The interview will be conducted as a guided conversation. I have a list of topics and general questions that I will go through, but your responses will guide the interview.

Thank you again for your participation. Does what I outlined sound okay? Do you have any questions before we begin? **START RECORDING**

Guiding Questions

1. Briefly describe your level of comfort in implementing a watchful waiting approach to diagnostic imaging in patients with low back pain.
    a. What are some of the challenges you face? *(Probe for patient satisfaction concerns, trust, communication barriers, diagnostic uncertainty, etc.)*
    b. In your opinion, what are the benefits of watchful waiting for diagnostic imaging?

2. Please tell us about your experiences with patients who have requested low-value spinal imaging for back pain. *(Probe for negative and positive experiences, successful ways of implementing watchful waiting, outcomes after ordering imaging, etc.)*

3. What do your patients need to know to better prepare them to partner with you to get the right care for their back pain? *[Share responses from patient focus group(s) and ask for clinician feedback, if available]*

4. What do clinicians need to know to better prepare them to deliver watchful waiting messages to their patients with back pain? *[Share responses from patient focus group(s) and ask for clinician feedback, if available]*

5. As I mentioned, we are designing an educational intervention for clinicians to deliver a watchful waiting message to patient requesting low-value imaging. Clinicians will be randomized to either the intervention or control group. Clinicians in each group will have scheduled visits with standardized patients over a nine-month period. Visits during the initial three months of the study will include educational content addressing patient-clinician communication. Each visit with the standardized patient will last no longer than 20 minutes.
    a. What are some key elements that should be incorporated into training clinicians?
    b. What are some effective ways to train clinicians to adapt new communication tools?
    c. What would make clinicians receptive to a training intervention of this nature? What are some drawbacks of this intervention? *(Probe for strengths of the proposed intervention as well as any concerns)*
    d. Are there aspects of the intervention that you believe might need to be more specifically targeted to your practice setting? Or specific types of patients you see?
    e. Can you please comment on the workflow and feasibility of the proposed intervention in the setting where you work?

6. Is there anything else you would like to add to our discussion?
